# The Archives of Dermatology

**Published:** 1876-04

**Authors:** 


*The Archives of Dermatology: A Quarterly Journal of Skin and Venereal Diseases.* Edited by L. DUNCAN BULKLEY, A. M., M. D. Volume First. New York: G. P. Putnam's Sons. 1875.

The first number of this journal appeared in October, 1874, under the able editorial charge of Dr. L. D. Bulkley, who as a writer on skin diseases has a wide reputation. The "*Archives*," the editor tells us in the first number, will be prepared with a view of meeting the wants of the general practitioner. Its general character will be a practical one. This promise has been well earried out in the first volume, and there is excellent promise of its further fulfilment in the second.

The subject matter of each issue is arranged under the following heads: Original Communications, Transactions of the New York Dermatological Society, Clinical Reports, Extracts and Translations, Digest of Dermatological Literature, Reviews and Book Notices, Correspondence and Miscellanies. Each of these departments is well supported. The Digest of Dermatological Literature is quite comprehensive in its scope, embracing more than would be supposed by those who have not had an opportunity of consulting its pages. In the number for April, 1875, for instance, we notice in the Digest, under Skin Diseases. considerations concerning their anatomy, physiology and pathology, acute and contagious inflammations, hypertrophies, atrophies and new formations, hemorrhages and neuroses; under Syphilis and Venereal Diseases, general questions in syphilis, nervous and visceral syphilis, and syphilis of the mouth, throat and larynx. Under each of the above heads a large number of articles, both American and foreign are referred to either by title simply, or *in extenso*, and the volume forms almost a complete bibliography of these subjects for the year.

We congratulate Dr. Bulkley and his co-workers and wish them continued success.

